# Objective Understanding of Front-of-Package Nutrition Labels among Nutritionally At-Risk Individuals

**DOI:** 10.3390/nu7085325

**Published:** 2015-08-24

**Authors:** Pauline Ducrot, Caroline Méjean, Chantal Julia, Emmanuelle Kesse-Guyot, Mathilde Touvier, Léopold K. Fezeu, Serge Hercberg, Sandrine Péneau

**Affiliations:** 1 Equipe de Recherche en Epidémiologie Nutritionnelle, Centre de Recherche en Epidémiologie et Statistiques, Université Paris 13, Inserm (U1153), Inra (U1125), Cnam, COMUE Sorbonne Paris Cité, Bobigny F-93017, France; E-Mails: c.mejean@eren.smbh.univ-paris13.fr (C.M.); c.julia@eren.smbh.univ-paris13.fr (C.J.); e.kesse@eren.smbh.univ-paris13.fr (E.K.-G.); m.touvier@eren.smbh.univ-paris13.fr (M.T.); l.fezeu@eren.smbh.univ-paris13.fr (L.K.F.); s.hercberg@eren.smbh.univ-paris13.fr (S.H.); s.peneau@eren.smbh.univ-paris13.fr (S.P.); 2 Département de Santé Publique, Hôpital Avicenne, Bobigny Cedex F-93017, France

**Keywords:** food labeling, front-of-package nutrition label, objective understanding, population at risk

## Abstract

In the ongoing debate about front-of-package (FOP) nutrition labels, little data exist regarding nutritionally at-risk populations, although they are critical targets of prevention programs. This study aimed to compare the impact of FOP labels on the ability to rank products according to their nutritional quality among French adults potentially at risk of poor dietary quality (*N* = 14,230). Four labels were evaluated: Guideline Daily Amounts (GDA), Multiple Traffic Lights (MTL), 5-Color Nutrition Label (5-CNL), Green Tick (Tick), along with a reference without label. Mixed models were used to assess how individual characteristics and FOP labels were associated with the ability to rank products. Older participants and those with a lower educational level, income, nutritional knowledge, and likelihood of reading nutrition facts were less skilled at ranking food products according to nutritional quality. Compared with individual characteristics, nutrition labels had an increased impact on food product ranking ability. Overall, 5-CNL corresponded to the highest rate of correct responses, followed by MTL, GDA, and Tick (*p* < 0.0001). The strongest impact of 5-CNL was observed among individuals with no nutritional knowledge (odds ratio (OR): 20.24; 95% confidence interval (CI): 13.19–31.06). Therefore, 5-CNL appeared to be effective at informing consumers, including those who are nutritionally at-risk, about the nutritional quality of food products.

## 1. Introduction

In the current fight against chronic diseases, promoting a healthy diet is a major objective of public health policies around the world [1]. One possible strategy for promoting a healthy diet is to encourage healthier food choices at the point of purchase [2]. However, in many western countries, the nutritional information currently displayed on food packages is generally difficult to read and interpret [3]. Thus, efforts should be made to provide simple and easily comprehensible information in order to enable consumers to make informed choices. For this reason, front-of-package (FOP) nutrition labeling is of major interest, since it increases consumer awareness of the nutritional quality of food [3,4,5,6,7,8].

Existing nutrition labels can be divided into two main categories: nutrient-specific labels and summary labels. Nutrient-specific labels display the amount of nutrients for which individual intake should be limited (e.g., fat, sodium). Examples of such labels include the Multiple Traffic Lights (MTL) used in the United Kingdom (UK) [9], and the Guideline Daily Amounts (GDA) used in the United States and in European countries, [10] now replaced by Reference Intakes (RI) in Europe [11]. In turn, summary labels provide the customer with an overall estimate of the nutritional quality of the product. Examples of such labels include simple formats, such as the Keyhole used in Scandinavian countries and applied only to healthy food products [12], and graded formats such as Guiding Stars used in North America, which provides a rating from zero to three stars according to the nutritional quality of the product [13].

A theoretical framework was proposed by Grunert *et al.*, to describe consumer food choice mechanisms when confronted with nutrition labeling [5]. First, the label should retain consumer attention. Next, for use, the label must be accepted and understood. These steps are potentially influenced by the label format, but also by individual-level determinants such as age, educational level, and interest in and knowledge of nutrition [5]. Evidence in the literature suggests that color-coded labels were more effective at focusing consumer attention and were preferred by individuals of low socioeconomic status, low educational level and poor nutritional knowledge [3,4,5,6,14,15,16]. In addition, summary labels may be more helpful in guiding vulnerable consumers toward healthier food choices, as they are more easily understood compared with nutrient-specific labels [7,8,17,18,19,20,21]. However, recent reviews reported a lack of research with subgroups of the population who might be at increased risk of consuming a lower-quality diet and/or among those who are overweight or obese [3,6,7,8]. Such vulnerable population subgroups include the elderly [22], those of lower socio-economic status, lower educational level [23], and lower interest in and knowledge of nutrition [24]. To date, most studies assessing consumer understanding have been performed on small samples, hence preventing accurate evaluation of label impact across subgroups [14,18,25]. In addition, many of those studies used subjective measures of consumer understanding [14,19] or performed objective measurements based on the comparison of only two products, potentially leading to random responses [17,18,25,26,27].

Finally, only a few studies have evaluated the understanding of a graded summary label, whereas recent reviews have emphasized its promising effects in real-world settings [7,8]. Therefore, in the context of the ongoing debate about the most effective labeling format, it is important to compare the understanding of different FOP nutrition labels, including a graded format, in subgroups potentially at risk of making poor food choices and consuming a lower-quality diet.

The main purpose of this study which used a sample of French adults was to identify individuals with a reduced ability to rank products according to nutritional quality. In addition, the influence of FOP labels on consumers’ ability to rank products according to nutritional quality was also assessed. Finally, the performance of each of four different FOP labels among nutritionally at-risk individuals was evaluated. The tested labels comprised two nutrient-specific formats (GDA and MTL) and two summary formats (Green Tick (Tick) and 5-Color Nutrition Label (5-CNL)).

## 2. Materials and Methods

### 2.1. Population

The NutriNet-Santé study is an ongoing web-based prospective cohort study launched in France in May 2009 with a scheduled follow-up period of 10 years. It aims to investigate the relationship between nutrition and chronic disease risk, as well as determinants of dietary behavior and nutritional status. The study was implemented in the general French population (Internet-using adult volunteers, aged ≥ 18 years). The rationale, design and methodology of the study have been published elsewhere [28]. In brief, to be included in the study, participants complete a baseline set of self-administered Web-based questionnaires assessing dietary intake, physical activity, anthropometric characteristics, lifestyle, socioeconomic conditions, and health status. As part of the follow-up, participants are asked on an annual basis to complete the same set of questionnaires. In addition, participants receive monthly email invitations to complete questionnaires about determinants of eating behavior, health status, *etc.* The study is conducted in accordance with the Declaration of Helsinki and all procedures were approved by the Institutional Review Board of the French Institute for Health and Medical Research (IRB Inserm n°0000388FWA00005831) and the *Commission Nationale de l*’*Informatique et des Libertés* (CNIL n°908450 and n°909216). All participants provide informed consent with an electronic signature. This study is registered in EudraCT (n°2013-000929-31).

### 2.2. Socio-Demographic Characteristics and Purchasing Habits Data

At baseline and annually, thereafter, participants in the NutriNet-Santé study are asked to provide socio-demographic data, including sex, age (18–30, 30–50, 50–65, >65 years), educational level (up to secondary, some college or university degree), and income (<1200 €, 1200–1800 €, 1800–2700 € and >2700 € per consumption unit). Monthly household income is calculated per “consumption unit” (CU), where one CU is attributed for the first adult in the household, 0.5 CU for other persons aged 14 or older, and 0.3 CU for children under 14, following national statistics methodology and guidelines [29]. For each participant, the most up-to-date available socio-demographic data were used. 

With regard to nutritional knowledge, participants were asked to self-estimate and report their level by choosing one of four options, ranging from “I know quite a bit about nutrition” to “I don’t know anything about nutrition”.

Purchasing habits data comprised information about frequency of reading the ingredient lists and/or nutrition facts (always, often, sometimes, never) on the packages, as well as grocery shopping frequency (always, often, sometimes, never).

### 2.3. Design

#### 2.3.1. Procedure

**Figure 1:**
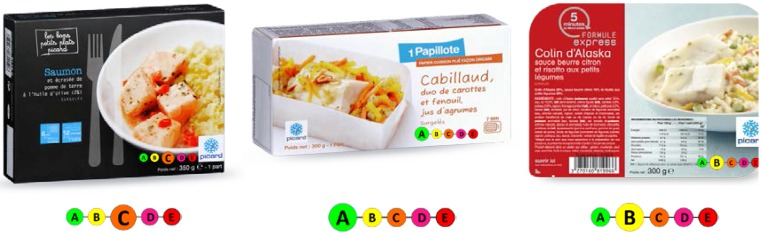
Screenshot of the stimulus material used in the study.

Objective understanding of the different FOP labeling formats was assessed in July 2014 via a Web-based questionnaire, under five different conditions: four alternatives corresponding to the four different FOP label formats and one alternative with no label. Subjects were asked to rank three products belonging to the same food category (e.g., Figure 1) according to their nutritional quality. Specifically, participants were shown pictures of the three products, each featuring the respective FOP label, and were asked: “From your point of view, please rank these products according to their nutritional quality”. For the ranking, participants could choose among the following options: “lowest nutritional quality”, “intermediate nutritional quality”, “highest nutritional quality”, or “I don’t know”. The three products were selected based on their differing nutritional quality, thus enabling ranking via the labels (except for the Tick format which enabled distinguishing only the top quality product). No other information on nutritional facts was provided and all quality labels (e.g., organic certification) were removed from the product images.

Five different product categories were tested: frozen prepared fish dishes, fresh pizzas, regular dairy products (mixed yogurts, cottage cheese and Greek yogurt), muesli breakfast cereals (chocolate, fruits and dried fruits/nuts), and appetizers (crisps and peanuts). To avoid potential effects of the product category upon understanding of the FOP label (*i.e*., due to knowledge of specific products), each label was associated with all product categories. Each participant was shown five label/product combinations where all five FOP label conditions and five product categories were represented. A rotation system based on a Latin Square design was employed to ensure that an equal number of participants were shown each label/product category combination while controlling for potential order effect of the labels. Thus, a total of 25 different versions of the questionnaire were used. For example, one participant was shown the 5-CNL on frozen prepared fish dishes, and MTL on fresh pizzas, while another participant was shown the 5-CNL on fresh pizzas and MTL on dairy products, *etc.* In addition, one respondent would be shown the 5-CNL first, while another participant would be shown the MTL first, *etc.*

Ranking was considered correct if the three products were ranked in the expected order (*i.e*., according to information on nutritional quality provided by the labels). Ranking was considered as incorrect if at least one mistake was made, or if the answer “I don’t know” was given. Expected ranking was the same whatever the situation.

#### 2.3.2. Label Formats

**Figure 2:**
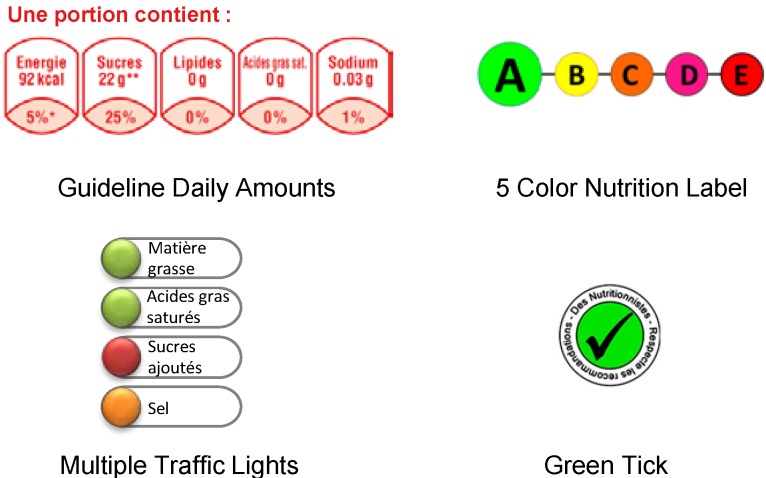
Front-Of-Package nutrition labels used in the study.

As noted above, four different label formats (Figure 2), providing varying levels of information about the products’ nutritional quality, were tested in the study. In the introduction to the questionnaire, the different label formats were presented and briefly explained to the participants.

Nutrient-specific formats:

1. Guideline Daily Amounts (GDA): this label indicates the kilocalories and the amount of total fat, saturated fatty acids, sugars, and sodium in grams per portion, as well as the corresponding contribution (in percentages) to the guideline-based daily intakes of these nutrients [10]. This label can be found on most food packaging on the French market, following a voluntary initiative on part of manufacturers. The GDA information was calculated by using the Food and Drink Federation’s guiding principles and was based on the average nutrient requirements for an adult woman.
2. Multiple Traffic Lights (MTL): this label, introduced by the Food Standards Agency (FSA) in the UK, provides an evaluation of the nutrient content regarding total fat, saturated fatty acids, sugars, and sodium. Depending on the quantity of the specific nutrient in the product (high, medium, low), a color is attributed to each nutrient (red, amber, green) indicating the nutritional quality of the product. Healthier food products feature more green and fewer red codes. The colors reflect the concentration in grams per 100 g (or 100 mL) of product, and the criteria of the FSA were applied to assign the color codes [9].

Summary formats:

3. Green Tick label (Tick): this label was derived from the “Keyhole” and “Pick the Tick” symbols, developed by the Swedish Food Administration and the Heart Foundation in Australia and New Zealand, respectively [12,30]. It reflects the overall nutritional quality of the food item and appears only on the healthier products within a food family. The Tick label was attributed to products assigned to the green or yellow categories by the 5-CNL (described below), and corresponds to “healthier” categories, as defined by the Office of Communication (OfCom) for advertising regulation [31].
4. Five-Color Nutrition Label (5-CNL): this label has been proposed specifically for the French market to guide consumer food choices [32]. It is based on the FSA nutrient profiling system [31], used by the OfCom. An adaptation of the guidelines was used specifically for cheese and added fat. This label provides information about the overall nutritional quality of a given food item. The label is represented by a scale of five colors, from green for the highest nutritional quality category to red for the lowest nutritional quality category, with corresponding letters (from A to E) [32]. Depending on the FSA score for each food item, the 5-CNL was: “green” (−15 to −2 points), “yellow” (−1 to 3 points), “orange” (4 to 11 points), “pink” (12 to 16 points), and “red” (≥17 points) [32].

Reference:

5. No label: a situation without any FOP nutrition labels was used as reference.

### 2.4. Statistical Analysis

Analysis was performed in 2015 on participants who had completed the questionnaire on FOP labels. Participants who had responded “I don’t know” to more than two-thirds of the items were excluded from analysis. Chi-square tests were used to compare included and excluded subjects. 

Mixed models for correlated data were used to evaluate how nutrition label formats and individual-level characteristics were associated with the ability to rank the three products. Individual characteristics were sex, age, educational level, monthly income, perceived nutritional knowledge, frequency of reading nutritional facts on product packages, and grocery shopping frequency. Variables displaying a significance level of *p* < 0.15 in univariate models were retained for the multivariate model. Missing covariate data for educational level and income/CU were imputed using the multiple imputation method. Interactions between correlates of FOP label understanding were assessed. Due to significant interactions, the multivariate logistic regression model was used to perform stratified analyses according to the individual characteristics presented above. Sensitivity analyses were performed on the whole sample, without excluding any participants. All tests of significance were two-sided, and a *p*-value < 0.05 was considered significant. Statistical analyses were performed using SAS software (version 9.3; SAS Institute Inc., Cary, NC, USA).

## 3. Results

### 3.1. Characteristics of the Sample

A total of 15,002 participants completed the FOP questionnaire. Among them, 772 were excluded because they had responded “I don’t know” to more than two-thirds of the items, which left 14,230 participants available for analysis. Characteristics of included and excluded participants are presented in Table 1. Compared with excluded participants, included participants were more often women, younger, had a higher educational level and higher perceived nutritional knowledge; they were also more likely to read nutritional facts on food packages and to do grocery shopping. 

**Table 1:** Individual characteristics of included (*N* = 14,230) and excluded (*N* = 772) participants.

	Included *N* = 14,230		Excluded *N* = 772		*p* ^a^
	*N*	%	*N*	%	
**Sex**					
Men	3111	21.86	308	39.90	**<0.0001**
Women	11,119	78.14	464	60.10	****
**Age (year)**					
18–30	2121	14.72	13	1.68	**<0.0001**
30–50	5112	14.91	164	21.24	****
50–65	4903	35.92	336	43.52	****
>65	2094	34.46	259	33.55	****
**Educational level**					
Up to secondary	4335	30.46	354	45.85	**<0.0001**
Some college	4432	31.15	187	24.22	
University degree	5203	36.56	198	25.65	
Missing data	260	1.83	33	4.27	
**Monthly income per household unit (€/CU ^b^)**					
<1200	2089	14.68	112	14.51	0.25
1200–1800	3222	22.64	180	23.32	
1800–2700	3537	24.86	185	23.96	
>2700	3895	27.37	196	25.39	
Missing data	1487	10.45	99	12.82	
**Perceived nutritional knowledge level**					
High	1966	13.82	72	9.33	**<0.0001**
Medium	7618	53.53	305	39.51	
Low	4323	30.38	311	40.28	
No knowledge	323	2.27	84	10.88	
**Frequency of reading nutritional facts on product packages**					
Always	2913	20.47	149	19.30	**<0.0001**
Often	7079	49.75	269	34.84	
Sometimes	3766	26.47	265	34.33	
Never	472	3.32	89	11.53	
**Grocery shopping frequency**					
Always	7965	55.97	399	51.68	**<0.0001**
Often	4426	31.10	218	28.24	
Sometimes	1609	11.31	11	14.38	
Never	230	1.62	44	5.70	

^a^: *p*-values based on chi-squared test; CU ^b^: “consumption unit”. One CU is attributed for the first adult in the household, 0.5 CU for other persons aged 14 or older, and 0.3 CU for children under 14; Boldface indicates statistical significance (*p* < 0.05).

### 3.2. Influence of Individual Characteristics and FOP Labels on the Ability to Rank Products according to Nutritional Quality

Results showing the association of product ranking ability with individual characteristics and label formats are presented in Table 2. Regarding socio-demographic characteristics, the odds of correctly ranking products were greater in women, younger participants, and those with a higher educational level. In addition, participants in the two highest income categories performed better in ranking products according to their nutritional quality compared with those in the lowest income category. Participants with the highest perceived nutritional knowledge gave more correct responses on the product ranking task than did those in the reference group, whereas those with medium-level knowledge of nutrition did not differ from the reference group. Similarly, participants who less frequently read nutritional facts on product packages gave less correct responses on the ranking task than did the reference, whereas those who reported often reading nutritional facts did not differ from the reference.

In both univariate and multivariate models, the odds of ranking products correctly according to nutritional quality was increased for all FOP labels used, compared with the no-label reference situation. Among the four formats, 5-CNL performed best, followed by MTL and GDA and the Tick label. 

Odds ratios corresponding to the effect of FOP labels on ranking performance were far stronger than those corresponding to the effect of individual characteristics.

Sensitivity analyses performed on the whole sample, without exclusion of participants, showed similar trends.

**Table 2:** Univariate and multivariate analyses based on mixed models showing the association between Front-of-Package label formats and objective understanding (*N* = 14,230) ^a^.

	Univariate	*p*	Mutivariate	*p*
	OR (95% CI)		OR (95% CI)	
**FOP labels**				
No label	1		1	
5-CNL	12.07 (11.41–12.78)	**<0.0001**	12.61 (11.91–13.36)	**<0.0001**
MTL	8.38 (7.92–8.86)	**<0.0001**	8.71 (8.22–9.22)	**<0.0001**
GDA	7.47 (7.06–7.91)	**<0.0001**	7.74 (7.31–8.20)	**<0.0001**
Tick	2.34 (2.21–2.47)	**<0.0001**	2.36 (2.23–2.49)	**<0.0001**
**Sex**				
Men	1		1	
Women	1.22 (1.18–1.27)	**<0.0001**	1.12 (1.06–1.17)	**<0.0001**
**Age (year)**				
18–30	1		1	
30–50	0.92 (0.88–0.96)	**<0.0001**	0.88 (0.83–0.93)	**<0.0001**
50–65	0.69 (0.66–0.73)	**<0.0001**	0.64 (0.61–0.68)	**<0.0001**
>65	0.53 (0.50–0.56)	**<0.0001**	0.47 (0.44–0.51)	**<0.0001**
**Educational level**				
Up to secondary	1		1	
Some college	1.24 (1.19–1.29)	**<0.0001**	1.15 (1.10–1.21)	**<0.0001**
University degree	1.30 (1.25–1.35)	**<0.0001**	1.17 (1.11–1.22)	**<0.0001**
**Monthly income per household unit (€/CU ^b^)**				
<1200	1		1	
1200–1800	0.94 (0.90–0.99)	**0.033**	1.00 (0.94–1.07)	0.99
1800–2700	0.99 (0.94–1.04)	0.74	1.09 (1.02–1.16)	**0.0068**
>2700	0.99 (0.94–1.04)	0.65	1.11 (1.05–1.18)	**0.0008**
**Perceived nutritional knowledge level**				
High	1.00		1	
Medium	0.92 (0.88–0.96)	**0.0004**	0.99 (0.94–1.05)	0.84
Low	0.81 (0.77–0.85)	**<0.0001**	0.92 (0.86–0.98)	**0.0072**
No knowledge	0.69 (0.61–0.77)	**<0.0001**	0.81 (0.71–0.93)	**0.0022**
**Frequency of reading nutritional facts on product packages**				
Always	1		1	
Often	0.98 (0.94–1.02)	0.35	0.99 (0.94–1.04)	0.69
Sometimes	0.89 (0.85–0.93)	**<0.0001**	0.90 (0.85–0.95)	**0.0004**
Never	0.77 (0.70–0.85)	**<0.0001**	0.77 (0.68–0.87)	**<0.0001**
**Grocery shopping frequency**				
Always	1		1	
Often	1.03 (0.99–1.06)	0.12	1.00 (0.96–1.04)	0.95
Sometimes	0.94 (0.89–0.99)	**0.015**	1.02 (0.96–1.09)	0.56
Never	0.83 (0.73–0.94)	**0.0039**	0.93 (0.80–1.09)	0.38

^a^: The modeled probability was correct ranking of the three products according to their nutritional quality; CU ^b^: “consumption unit”. One CU is attributed for the first adult in the household. 0.5 CU for other persons aged 14 or older, and 0.3 CU for children under 14; Boldface indicates statistical significance (*p* < 0.05); OR:odds ratio; CI: confidence interval; FOP: front-of-package.

### 3.3. Comparison of Label Performance across Subgroups

Label performances across subgroups of individuals are presented in Table 3. Results show the same trend across subgroups, although odds ratios vary to differing extents. Compared to the reference situation (no label), all label formats significantly increased the ability of participants to correctly rank products according to nutritional quality. The 5-CNL was the label that had the best performance in all subgroups (lowest OR: 8.72, (95% CI: 7.46–10.18); greatest OR: 20.24 (95% CI: 12.59–31.06)), followed by MTL (OR range: 6.30 (5.39–7.35)–10.62 (9.15–12.33), GDA (OR range: 5.53 (3.99–7.66)–8.79 (7.92–9.76)) and Tick (OR range: 1.98 (1.72–2.29)– 2.64 (1.63–4.27)) labels.

Sensitivity analyses performed on the whole sample, with no exclusion of participants, revealed similar trends.

**Table 3:** Multivariate mixed model of the association between objective understanding and Front-of-Package labels, across subgroups at risk (*N* = 14,230) ^a^.

	No Label	5-CNL	MTL	GDA	Tick	*p* ^b^
		OR (CI 95%)	OR (CI 95%)	OR (CI 95%)	OR (CI 95%)	
**Sex**						
Men	1	13.44 (11.83–15.27)	9.33 (8.21–10.60)	7.45 (6.56–8.46)	2.55 (2.24–2.90)	**<0.0001**
Women	1	12.41 (11.63–13.24)	8.55 (8.02–9.12)	7.85 (7.36–8.37)	2.31 (2.17–2.46)	**<0.0001**
**Age (year)**						
18–30	1	14.20 (12.25–16.46)	10.62 (9.15–12.33)	8.56 (7.41–9.89)	2.28 (1.99–2.61)	**<0.0001**
30–50	1	15.36 (13.93–16.94)	9.74 (8.85–10.71)	8.05 (7.32–8.86)	2.39 (2.18–2.62)	**<0.0001**
50–65	1	11.50 (10.43–12.68)	8.03 (7.28–8.85)	7.36 (6.67–8.11)	2.32 (2.10–2.56)	**<0.0001**
>65	1	8.72 (7.46–10.18)	6.30 (5.39–7.35)	6.63 (5.68–7.74)	2.40 (2.04–2.82)	**<0.0001**
**Educational level**						
Up to secondary	1	9.91 (8.94–11.00)	7.30 (6.58–8.10)	7.03 (6.34–7.80)	2.44 (2.20–2.72)	**<0.0001**
Secondary	1	12.59 (11.36–13.96)	8.73 (7.88–9.66)	7.26 (6.55–8.05)	2.39 (2.16–2.64)	**<0.0001**
University	1	15.61 (14.17–17.19)	10.00 (9.09–11.01)	8.77 (7.98–9.64)	2.26 (2.06–2.48)	**<0.0001**
**Monthly income per household unit (€/CU ^c^)**						
<1200	1	11.99 (10.42–13.79)	8.37 (7.24–9.66)	7.22 (6.27–8.31)	2.33 (2.03–2.69)	**<0.0001**
1200–1800	1	12.46 (11.09–14.00)	8.79 (7.82–9.87)	7.59 (6.75–8.54)	2.54 (2.26–2.85)	**<0.0001**
1800–2700	1	11.84 (10.56–13.28)	7.89 (7.04–8.85)	7.48 (6.66–8.40)	2.12 (1.90–2.38)	**<0.0001**
>2700	1	14.06 (12.59–15.71)	9.77 (8.76–10.90)	8.54 (7.65–9.53)	2.47 (2.22–2.74)	**<0.0001**
**Perceived nutritional knowledge level**						
High	1	10.52 (9.05–12.22)	7.79 (6.71–9.04)	7.43 (6.40–8.62)	1.98 (1.72–2.29)	**<0.0001**
Medium	1	12.46 (11.53–13.47)	8.74 (8.08–9.45)	8.54 (7.89–9.24)	2.38 (2.21–2.57)	**<0.0001**
Low	1	13.70 (12.30–15.26)	9.15 (8.22–10.19)	6.87 (6.17–7.64)	2.52 (2.26–2.80)	**<0.0001**
No knowledge	1	20.24 (13.19–31.06)	9.80 (6.48–14.80)	6.56 (4.29–10.03)	2.55 (1.63–3.99)	**<0.0001**
**Frequency of reading nutritional facts on product packages**						
Always	1	10.32 (9.14–11.64)	8.00 (7.07–9.06)	8.02 (7.09–9.06)	2.23 (1.98–2.52)	**<0.0001**
Often	1	13.14 (12.10–14.26)	9.08 (8.37–9.86)	8.58 (7.90–9.31)	2.48 (2.29–2.69)	**<0.0001**
Sometimes	1	13.84 (12.33–15.53)	8.86 (7.91–9.92)	6.56 (5.85–7.34)	2.20 (1.97–2.46)	**<0.0001**
Never	1	11.93 (8.67–16.41)	7.07 (5.12–9.77)	5.53 (3.99–7.66)	2.60 (1.87–3.61)	**<0.0001**
**Grocery shopping frequency**						
Always	1	11.41 (10.58–12.32)	7.93 (7.36–8.56)	7.25 (6.72–7.82)	2.30 (2.14–2.48)	**<0.0001**
Often	1	14.69 (13.22–16.32)	9.68 (8.71–10.76)	8.79 (7.92–9.76)	2.39 (2.16–2.65)	**<0.0001**
Sometimes	1	14.06 (11.80–16.74)	10.47 (8.78–12.48)	7.89 (6.62–9.42)	2.55 (2.14–3.04)	**<0.0001**
Never	1	12.92 (8.06–20.70)	9.52 (5.92–15.31)	7.11 (4.50–11.23)	2.64 (1.63–4.27)	**<0.0001**

^a^ Model was adjusted for sex, age, educational level, monthly income, purchasing frequency, perceived nutritional knowledge and frequency of reading nutritional facts on product packages; ^b^ The modeled probability was a correct ranking of the three products according to their nutritional quality; ^c^ CU: consumption units. One CU is attributed for the first adult in the household. 0.5 for other persons aged 14 or older and 0.3 for children under 14; Boldface indicates statistical significance (*p* < 0.05); GDA: Guideline Daily Amounts; MTL: Multiple Traffic Lights; 5-CNL: 5-Color Nutrition Label; Tick: Green Tick; OR:odds ratio; CI: confidence interval.

## 4. Discussion

Results of the present study indicate that understanding of FOP labels differs across groups of individuals. The ability to rank products according to nutritional quality was lower in older subjects, men, participants with a lower educational level, lower income, lower nutritional knowledge, and those who were less likely to read nutrition facts on food packages. However, results of the present study also indicated that, compared to individual characteristics, nutrition labels had a stronger influence on food product ranking ability. The 5-CNL graded summary label was the format that was most easily understood in all subgroups, and it performed particularly well among participants with no nutritional knowledge.

### 4.1. Influence of Individual Characteristics and FOP Labels on the Ability to Rank Products according to Nutritional Quality

Results of the present study are in line with previous reports that found that some population subgroups display a decreased likelihood of understanding FOP nutrition labels [3,7,8,15,17,18]. In this study, women performed significantly better than did men. Women’s greater interest in nutrition [5], as well as the fact that they are more likely to use nutrition labels [3,4,5,33], might account for their increased ability to rank products according to nutritional quality. Data from the present study support previous studies indicating that older participants and those with lower educational level had more difficulty in understanding nutrition labels [14,15,17,26,34]. Similar to this study, two studies indicated that participants with lower self-reported nutritional knowledge or who less often read labels were less likely to accurately interpret FOP labels [14,18]. To our knowledge, no study has evaluated the impact of income on FOP label understanding. However, two studies reported that consumers belonging to lower socio-economic strata had more difficulty understanding nutrition labeling than did their more affluent counterparts [17,18]. Thus, the positive association between income and understanding of FOP labels, which was observed in the present study following adjustment for educational level, could be due to social status, potentially influencing the interest in healthy eating, and the ability to process information [15].

Overall, in the present study, participants displaying increased difficulty understanding nutrition labels had similar sociodemographic profiles to individuals displaying a reduced likelihood of using nutrition labels [3,4,7], and those potentially at-risk regarding their nutritional status [35,36,37,38,39,40,41]. Generally, a better understanding of labels promotes their use [42], thus, it appears crucial to select FOP labels that enable accurate understanding among vulnerable subgroups.

Consistent with previous work, results of the present study indicate that nutrition labels are efficient tools for increasing consumers’ ability to compare nutritional quality across food products, compared with a reference situation presenting no label [7,17,18,25,27,43]. Among the different label formats, 5-CNL performed best at enabling consumers to rank food products according nutritional quality, followed by MTL, GDA, and the Tick label, which supports the potential of a graded summary label suggested in recent reviews [7,8].

Interestingly, results of this study highlighted the fact that nutrition labels improve individuals’ ability to rank food products according to nutritional quality to a much greater extent (greatest OR: 12.64 (95%CI: 11.93–13.39)), than did individual characteristics (greatest OR: 1.17 (95%CI: 1.12–1.23)). This is of particular interest given that nutrition labeling is conceivably easier to modify than are individual factors. Among potentially-modifiable individual-level factors, nutritional knowledge and the frequency of reading nutrition facts on food packages could be improved by nutrition education [44,45]. However, such interventions are most effective when dispensed in childhood [46], with a potential impact over the long term. In turn, previous studies have suggested that introducing nutrition labels had the advantage of being cost-effective and could bring about substantial and relatively quick health benefits on the population level [47,48].

### 4.2. Comparison of Label Performance across Subgroups

Across subgroups of participants, the 5-CNL had the strongest positive association with participants’ ability to rank products, followed by MTL, GDA and Tick. This trend was observed especially in subjects with low nutritional knowledge. Although several reviews emphasized the potential of a graded label [7,8], consumer understanding of such a format has been poorly studied in the literature. To our knowledge, only one study found that graded labels were easier to understand than was the color-coded GDA among participants with poor nutritional knowledge and who never read labels [14]. However, no significant differences were observed between the summary graded label and MTL and label formats were similarly understood in individuals of various age and educational level [14]. A possible explanation for such discrepancies might be that the large sample size used in the present study provided sufficient statistical power for performing subgroup analyses. Thus, a more accurate comparison among label formats was possible. Next, two particular attributes might explain the better performance of 5-CNL compared with MTL. First, 5-CNL summarizes the product’s nutritional quality in a single indicator, thus preventing misunderstanding of nutrition terminology [4] and obviating the need to process and synthesize information on nutrient content. Second, the 5-CNL combined color and text, which has been shown to improve readability of FOP labels [6,7]. The 5-CNL outperformed all other formats across all nutritionally at-risk subgroups and performed particularly well among individuals with no nutritional knowledge. Thus, this format appeared to be a well-adapted tool for fairly informing consumers about the nutritional quality of foods. 

As regards the nutrient-specific label formats, and in agreement with previous research, results of the present study showed that the MTL was better than the GDA at increasing understanding [17,18,19,25,26]. Indeed, color-coded labels such as MTL have been shown to enhance consumer ability to evaluate product healthiness [7]. In turn, nutrition labels that include numbers and percentages, such as GDA, were found to be confusing to many consumers, particularly those with lower educational levels or literacy [3,6,7]. In contrast with these findings, results of a recent study performed among consumers from the UK, Germany, Poland, and Turkey showed that GDA and MTL labels including numerical nutritional information performed similarly to enable consumers to identify the healthier alternative within a set of three food products [49]. A possible explanation to account for such discrepancies may be that numbers displayed on the MTL have been confusing for participants. Although previous studies reported that simple summary formats are preferred by vulnerable subgroups [14,19,20,50], in the present study, the Tick label was associated with the poorest performance as regards consumer understanding. However, Malam *et al.* noted that the preference for a particular FOP labeling format did not necessarily indicate a better understanding of that label [18]. In addition, in a previous study, a simple summary label showed similar effect compared with other FOP labels (*i.e.*, GDA, MTL), at differentiating healthiness of three food products [49], suggesting that such a format might not necessarily be optimal when ranking more than two products according to their nutritional quality. One explanation to account for the poor performance of the Tick label might be that such a format enables consumers to identify healthier products, but not to differentiate between products of medium and low nutritional quality [51,52]. Thus, oversimplification might lead to a loss of information and to misinterpretation of the label [53,54].

### 4.3. Strengths and Limitations

A major strength of this study was its large sample size and the heterogeneity of socio-demographic profiles, enabling sufficiently-powered analyses across subgroups. Moreover, to limit desirability bias, an objective measure of participant understanding was used. A set of three products was used so as to be more realistic and to limit random responses. In addition, the potential effects of the labels’ order of appearance were controlled by using a rotation system. To limit the effect related to product choice, five food categories were included and all combinations of products/labels were tested.

This study is subject to several limitations. First, participants of the NutriNet-Santé study are volunteers in a nutrition-focused cohort. Compared with excluded participants, included participants had higher knowledge of nutrition and read nutrition facts more often. Indeed, compared with the present study, previous studies reported lower levels of nutrition facts reading among French consumers (*i.e*., 9%) [55] and lower knowledge of nutrition guidelines across a representative sample of French adults [56]. Caution is, therefore, needed when interpreting and generalizing the present results. However, interest in, and knowledge of, nutrition might have led to a greater number of correct responses in the reference situation (“no-label”) compared with the general French population, thus possibly decreasing the labels’ influence. In addition, the study was performed among Internet users, possibly leading to sociodemographic differences compared with the general French population [57]. However, the sample featured sociodemographic diversity, including individuals from typically under-represented subgroups in traditional surveys (older, low socio-economic status). Another limitation pertained to the fact that the assessment was not performed in a real-life environment, where a number of factors influence consumer understanding. Indeed, noise, marketing messages, time pressure, and the multitude of available products are likely to hinder label processing [58,59,60].

## 5. Conclusions

The present study is one of the first investigating label understanding in a large sample of general population-based volunteers, including individuals potentially at at-risk regarding their nutritional status. The results showed that the ability to compare products according to nutritional quality differed across groups of individuals and was lower in nutritionally at-risk individuals. The results also highlighted the fact that the impact of nutrition labeling on product comparison was stronger than were individual characteristics. These data bring new supportive evidence to the current debate on food labeling. In particular, the 5-CNL graded color-coded label displayed a strong performance across population subgroups. In addition, this graded label was shown to perform particularly well in participants with no nutritional knowledge. Introducing such a label would provide a relatively fair understanding of FOP labels among consumers, and might potentially encourage those who do not frequently use nutritional information to favorably change their behavior as regards food choices.
